# Response to a Rabies Epidemic, Bali, Indonesia, 2008–2011

**DOI:** 10.3201/eid1904.120380

**Published:** 2013-04

**Authors:** Anak Agung Gde Putra, Katie Hampson, Janice Girardi, Elly Hiby, Darryn Knobel, Wayan Mardiana, Sunny Townsend, Helen Scott-Orr

**Affiliations:** Disease Investigation Center, Denpasar, Bali, Indonesia (A.A.G. Putra);; College of Medical, Veterinary and Life Sciences, University of Glasgow, Glasgow, United Kingdom (K. Hampson, S. Townsend);; Bali Animal Welfare Association, Ubud, Bali (J. Girardi); World Society for the Protection of Animals, London, United Kingdom (E. Hiby);; Faculty of Veterinary Science, University of Pretoria, Onderstepoort, South Africa (D. Knobel);; Bali Province Livestock Services, Denpasar, Bali (I.W. Mardiana);; Faculty of Veterinary Science, University of Sydney, Camden New South Wales, Australia (H. Scott-Orr)

**Keywords:** rabies, epidemic, Indonesia, Bali, rabies vaccination, dogs, dog bites, zoonoses, viruses

## Abstract

Emergency vaccinations and culling failed to contain an outbreak of rabies in Bali, Indonesia, during 2008–2009. Subsequent island-wide mass vaccination (reaching 70% coverage, >200,000 dogs) led to substantial declines in rabies incidence and spread. However, the incidence of dog bites remains high, and repeat campaigns are necessary to eliminate rabies in Bali.

Rabies was first reported in Indonesia in 1884 and now occurs in 24 of the country’s 33 provinces (*1*–*3*). On Bali Island, the first cases of rabies in humans and dogs were confirmed in 2008 on Bukit Peninsula (Figure). Despite control efforts in 2008–2009, rabies spread across the island. In the following 3 years, >130 persons died from rabies (primarily persons who did not receive postexposure prophylaxis [PEP]) (*4*), and PEP was given to >130,000 persons with dog bites. This outbreak resulted in considerable fear and anxiety and cost >US $17 million. We report on the outbreak progression and the effect of initial and subsequently improved control measures.

**Figure:**
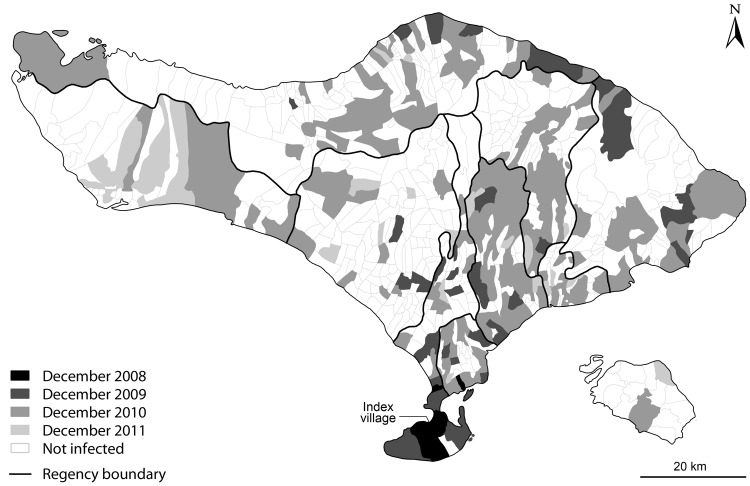
Timing of confirmed rabies cases in villages across Bali since the first case was confirmed on the island in November 2008. Darker shading indicates earlier detection according to the months since the first case was detected in the index village (marked), lighter shading indicates later detection, and white shading indicates no detected cases by December 2011.

## The Study

When the 2008 Bali rabies outbreak began, the island had no policies for rabies PEP and no dog bite surveillance, rabies diagnostic facilities, or vaccines for dogs. In response to the outbreak, the Indonesian government provided Bali with postexposure rabies vaccine for humans (Verorab), for intramuscular administration according to World Health Organization guidelines, and vaccines for dogs (*10*). The Australian government helped establish a direct fluorescent antibody (DFA) test at the Disease Investigation Center, Denpasar, Bali, and provided supplies for emergency dog vaccination. Surveillance was implemented by DFA testing of brain specimens from dogs that died or were killed after showing signs of rabies and from culled dogs. This surveillance, although imperfect, proved critical in tracking rabies spread (Figure).

In Bali, the first 2 regencies (administrative divisions below provincial governments) affected by the rabies outbreak were Denpasar and Badung. In December 2008, the Balinese government began culling (using strychnine-laced baits or blow darts) unconfined dogs in areas of Denpasar and Badung with confirmed rabies cases and began vaccinating dogs at fixed posts. The locally manufactured vaccine required a booster after 3 months. It was estimated from a survey in Badung, where the human:dog ratio was 8.3:1 (*5*), that 40% of dogs in Badung and Denpasar were vaccinated during December 2008–March 2009 and that 25% received booster vaccinations by June 2009. Over 90% of the dogs in Bali are owned, but most are free-roaming and hard to catch for 1 parenteral vaccination, let alone booster vaccinations (*6*,*7*). Thus, the emergency response failed to contain the outbreak, and by September 2010, rabies had been confirmed in 221 (30.5%) villages throughout Bali (Table; Figure).

**Table:** Indicators of rabies incidence and spread among the human, dog, and other animal populations before and during mass island-wide dog vaccination campaigns, Bali, Indonesia, 2008–2011*

Indicator	Before campaign, Nov 8–Sep 10, 2008	During 1st campaign, Oct 10, 2010–Apr 11, 2011	During 2nd campaign, May 11–Dec 11, 2011
Observation period, mo	22	7	8
Average no. rabid dogs/mo	45	11	6
Average apparent monthly attack rate among dogs, %†	0.03	0.01	0.01
Total no. villages with cases detected among dogs	221	269	282
No. villages with newly detected cases	NA	48	13
Rate of spread, no. villages with newly detected dog cases/mo	10	6.8	1.6
Remaining known villages with cases among dogs, no. (%)	140 (19.4)	48 (6.6)	30 (4.1)
No. dog bites treated/mo (bites/day)	6,256 (208)	4,589 (153)	4,197 (140)
Human deaths	94	34	9
Estimated no. culled dogs	107,900	40,500	14,000
No. dogs vaccinated (estimated coverage, %)	>73,000 (40)‡	249,429 (>70)	231,155 (>70)

*Rabid dogs correspond to cases confirmed by using the direct fluorescent antibody test. Villages were classified as free from rabies if no cases were detected for at >6 mo. Coverages were initially estimated from human: dog ratios and subsequently from observations of the proportion of dogs with collars indicating vaccination. The number of culled dogs was also estimated because some culling was carried out by communities rather than by government. NA, not applicable. †Attack rate, confirmed rabid dogs divided by estimated unvaccinated dog population. ‡§Data were not available for dogs vaccinated and boosted during the first few months of the outbreak; therefore, only data on vaccinations in Gianyar and Bangli Regencies are shown.

In 2009, the Australian Government donated long-lasting vaccines for dogs, but operational funds for administration were unavailable. A local nongovernment organization, the Bali Animal Welfare Association (BAWA), developed a technique to improve vaccination coverage by training teams to catch dogs with nets. During December 2009–July 2010, 6-person BAWA teams using this technique piloted door-to-door vaccinations throughout Gianyar Regency, where BAWA is based. The teams vaccinated 48,000 dogs in Gianyar and 25,000 in nearby Bangli Regency. The World Society for the Protection of Animals donated supplies for this pilot, and BAWA covered operational costs. Surveys of collared (vaccinated) dogs on consecutive days after vaccinations indicated 70% coverage in almost all banjars (subvillages). Beginning in October 2010, BAWA teams and Balinese government staff worked together, with funding from the World Society for the Protection of Animals, to vaccinate dogs throughout most of Bali, subject to the official suspension of culling. By April 2011, a total of 249,429 dogs had been vaccinated, with coverage >70% in most banjars. During this campaign, dogs in Gianyar Regency were revaccinated because 18 months had passed since the pilot and coverage had declined because of population turnover and movement. A second island-wide campaign using these methods was completed in December 2011 by the Balinese government, coordinated by the Food and Agriculture Organization, and achieved similar coverage (Table).

During 2010, rabies was confirmed in 417 dogs, 2 cats, and 3 cows. Of the 417 dogs, 387 (93%) were probably unvaccinated; 30 had reportedly been vaccinated, but only 9 had a clear vaccination date; 5 were positive for rabies shortly after vaccination and were likely incubating the disease when vaccinated; and 4 cases were considered vaccination failures.

When the first island-wide vaccination campaign began in 2010, a total of 140 (19.4%) villages still reported rabies (>1 case in the previous 6 months), and 81 (11.2%) villages that previously reported cases were considered rabies-free (no cases detected for >6 months). In addition, during this island-wide campaign (October–April 2011), rabies was detected in 48 previously rabies-free villages. By December 2011, only 30 (4.1%) villages were not considered rabies-free (Table). Before island-wide vaccination, rabies was detected in 10 new villages per month; during the first and second island-wide vaccinations, rabies was detected in 6.8 and 1.6 villages per month, respectively. The monthly number of confirmed cases before mass vaccination was also much higher (44.7 cases) than during the first (10.8 cases) and second (6.0 cases) mass vaccination campaigns, and concomitantly, the island-wide attack rate (confirmed rabid dogs per estimated unvaccinated population) declined from 0.027% to 0.01% (Table). Reported dog bites declined slowly, from 6,256 bites per month before island-wide vaccination to 4,589 and 4,197 bites per month during the first and second vaccination campaigns, respectively. However, human deaths from rabies declined from 94 (4.3/month) before island-wide vaccination to 34 (4.8/month) during the first campaign (24/34 persons were bitten during the prevaccination period) to 9 (1.1/month) deaths during the second campaign.

## Conclusions

Rabies was detected in Bali in 2008; it was probably brought by fishermen from the island of Sulawesi (Indonesia), as happened on the island of Flores (Indonesia) (*3*), and subsequently spread throughout the island. Early containment attempts by limited fixed-point dog vaccination and culling were unsuccessful. This was likely due to insufficient funding, largely inaccessible free-roaming dog populations with high turnover, limited availability of long-lasting dog vaccines (and means to identify vaccinated dogs), and inconsistent cold chains.

These issues were gradually addressed, and island-wide vaccinations in 2010 and 2011 approached the recommended target of 70% coverage (*8*,*9*); postvaccination surveys of collared dogs enabled better coverage estimates. Considerable coordination was required among Bali’s provincial and regency governments, which was facilitated through training and data management systems. Nonetheless, reporting remained challenging due, in part, to limited infrastructure.

Vaccination campaigns reduced rabies incidence and spread, resulting in decreased attack rates at the regency level and island-wide. In contrast, culling was ineffective in suppressing rabies and can be counterproductive (*10*). Although panic led to demand for culling in some locations, many communities objected because of religious beliefs and, especially, when owned (often vaccinated) dogs were culled. New puppies were brought to replace culled dogs, and some dogs were moved to avoid culls, possibly resulting in the transportation of infected dogs. Incidence declines due to vaccinations reduced the public health threat and panic that triggered culling; ≈108,000 dogs were culled before island-wide vaccination, compared with 40,000 during the 2 vaccination campaigns. However, long-term acceptable dog population control is still sought on Bali.

DFA testing proved an effective surveillance method; dog bites were a less sensitive measure. The incidence of reported bites is higher on Bali than in Indonesian provinces where rabies is endemic; this may reflect heightened awareness about rabies or be related to the high densities of humans and dogs. Rabid dogs generally bite without provocation and die <10 days after clinical signs develop (*11*); thus, a short observation period (*12*) may allow more judicious PEP administration but is often impractical with unrestrained dogs.

Mass dog vaccinations substantially reduced rabies incidence on Bali and must be continued if elimination is to be achieved. Further research is needed to assess how many more campaigns are needed. Improved surveillance and control of inter-island dog movement are necessary to prevent further rabies spread within Indonesia.
